# Differential Effects of *Mycobacterium bovis* BCG on Macrophages and Dendritic Cells from Murine Spleen

**DOI:** 10.3390/ijms161024127

**Published:** 2015-10-13

**Authors:** Zhengzhong Xu, Chuang Meng, Bin Qiang, Hongyan Gu, Lin Sun, Yuelan Yin, Zhiming Pan, Xiang Chen, Xinan Jiao

**Affiliations:** Jiangsu Key Laboratory of Zoonosis/Jiangsu Co-Innovation Center for Prevention and Control of Important Animal Infectious Diseases and Zoonoses, Yangzhou University, Yangzhou 225009, China; E-Mails: zzxu@yzu.edu.cn (Z.X.); mengchuangyzu@163.com (C.M.); qiangdabin@163.com (B.Q.); ghygrace@163.com (H.G.); sunlin@yzu.edu.cn (L.S.); yylan@yzu.edu.cn (Y.Y.); zmpan@yzu.edu.cn (Z.P.)

**Keywords:** BCG, macrophage, dendritic cell

## Abstract

Macrophages (MΦ) and dendritic cells (DCs) are both pivotal antigen presenting cells capable of inducing specific cellular responses to inhaled mycobacteria, and thus, they may be important in the initiation of early immune responses to mycobacterial infection. In this study, we evaluated and compared the roles of murine splenic DCs and MΦs in immunity against *Mycobacterium bovis* Bacillus Calmette-Guérin (*M.bovis* BCG). The number of internalized rBCG-GFP observed was obviously greater in murine splenic MΦs compared with DCs, and the intracellular reactive oxygen species (ROS), inducible nitric oxide synthase (iNOS) and nitric oxide (NO) levels in MΦs were all higher than in DCs. DCs have a stronger capacity for presenting Ag85A peptide to specific T hybridoma and when the murine splenic MΦs were infected with BCG and rBCG::Ag85A, low level of antigen presenting activity was detected. These data suggest that murine splenic MΦs participate in mycobacteria uptake, killing and inducing inflammatory response, whereas the murine splenic DCs are primarily involved in specific antigen presentation and T cell activation.

## 1. Introduction

One of the major causes of death by infectious disease worldwide is *Mycobacterium tuberculosis* (*M.tb*) infection, which is an extraordinarily successful human pathogen [1]. Control of *M.tb* is mainly the result of productive teamwork between T-cell populations and antigen presenting cells. CD4^+^ T cell responses are crucial to the control of *M.tb* infection in humans and various species of animals [2,3], and require processing and presenting of *M.tb* Ags to generate peptide-MHCII complexes [4].

Both macrophages (MΦ) and dendritic cells (DCs) have been suggested be important in the defense against *M.tb* infection, they all participate in the initiation of early innate immune responses to mycobacterial infections, and they can also possess and present mycobacterial antigen to CD4^+^ T cells to trigger specific cellular responses. However, DCs are a group of cells that are specialized for the antigen presentation to T cells, and since the major role of MΦs is the rapid ingesting and killing of the invading organism, they must have different roles and cooperate closely in the defense against *M.tb*.

Once inhaled, *M.tb* is readily phagocytosed, processed, and presented by antigen presenting cells. A serial of microbicidal mechanisms will be activated after the MΦs were infected by *M.tb*, including phagolysosome fusion, respiratory burst, and the secretion of multiple proinflammatory cytokines, which control the growth of inhaled mycobacteria and activate additional recruited immune cells [5,6]. Although both MΦs and DCs can process and present Ag to T cells, DCs are unique because of their role in priming the T cell response against many pathogens [7,8]. Initiation of the CD4^+^ T cell response to the mycobacterial antigen ESAT-6 is likely to be critically dependent on DCs, and MΦs may be sufficient to activate memory T cells [7].

Since many *in vitro* studies about immune responses against mycobacterial infection of antigen presenting cells (APCs) use human or mouse cell lines, and bone marrow derived and peripheral blood derived cell types under different experimental conditions, it is not sure whether the conclusions hold in general. Further, studies *in vivo* present a more complex situation in which to evaluate the effects of APCs against mycobacterial infection. For example, experiments *in vivo* cannot distinguish the small proportion of infected APCs from the large proportion of uninfected APCs [9].

As a result, the murine splenic DCs and MΦs were sorted from mice as a cell model in this study. We investigated the interactions of *M.bovis* BCG with DCs and MΦs, and compared their different roles in immunity against *M.bovis* BCG infection. We have examined the ability of murine MΦs and DCs to internalize *M.bovis* BCG, then analyzed the kinetics of cytokine gene expression and antigen presenting activity of *M.bovis* BCG-infected murine splenic DCs and MΦs. Further study of the mechanisms may enhance our ability to prime innate and adaptive immunity, and advance the development of improved BCG-based immunization strategies to control TB.

## 2. Results

### 2.1. Infection of Murine Splenic MΦs and DCs with rBCG-GFP

**Figure 1:**
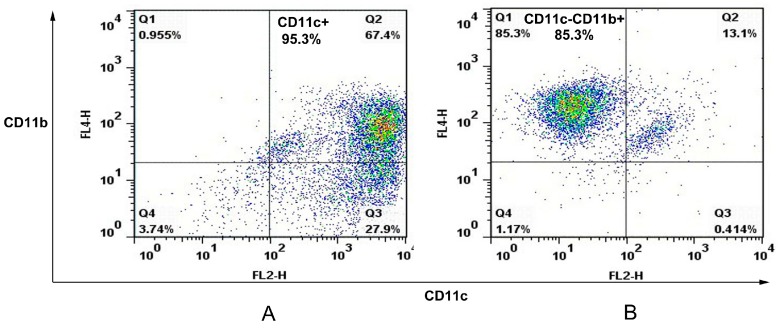
Cell sorting of murine splenic MΦs and DCs. In this case, the single spleen cell suspension of C57/BL6 mice was first stained with anti-CD11c Microbeads before autoMACS separation led to a positive cell sample of CD11c^+^ cells (Q2 and Q3) with purity of 95.3% (**A**); The negative fraction was further incubated with anti-CD11b Microbeads giving a positive cell fraction containing CD11c^−^CD11b^+^ cells (Q1) with purity of 85.3% (**B**). Q1: CD11c^−^CD11b^+^, Q2: CD11c^+^CD11b^+^, Q3: CD11c^+^CD11b^−^, Q4: CD11c^−^CD11b^−^.

**Figure 2:**
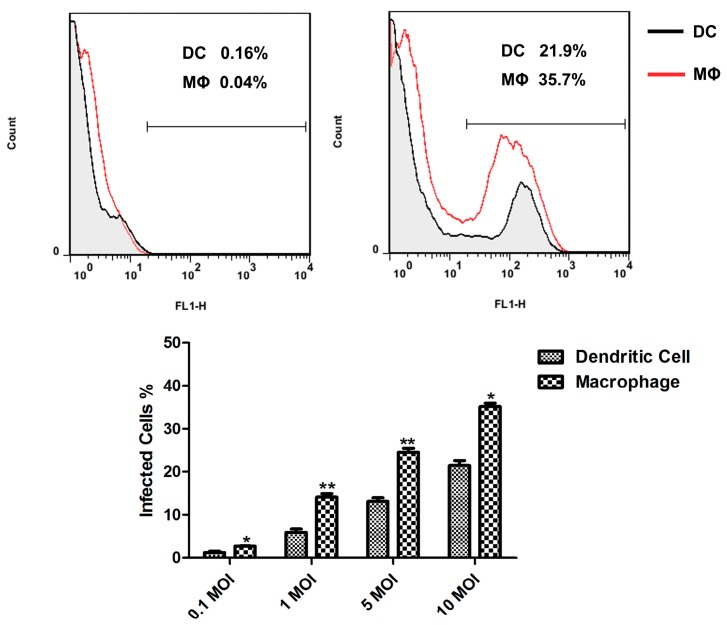
Infection of murine splenic MΦs and DCs with rBCG-GFP. The percentage of dendritic cells and macrophages infected by rBCG-GFP was determined. Data depicted represent the mean values ± SEM. Statistical significance was determined by a Student’s *t*-test (* *p* < 0.05, ** *p* < 0.01).

To investigate the ability of murine MΦs and DCs to internalize *M.bovis* BCG, the rBCG-GFP was used to infect sorted murine splenic MΦs and DCs *in vitro* (Figure 1), and the infected rate of murine MΦs and DCs was detected. The percentages of infected cells were measured by flow cytometry (FCM) 6 h after infection, they were 1.23% ± 0.20%, 6.40% ± 1.01%, 13.91% ± 1.45% and 22.03% ± 1.25% for DCs, and 2.59% ± 0.39%, 13.01% ± 2.11%, 25.20% ± 1.26% and 31.07% ± 1.47% for MΦs at MOI values of 0.1, 1, 5 and 10, respectively (Figure 2). A significant difference was observed in the number of ingested rBCG-GFP, which was higher in murine splenic MΦs compared with DCs, and the mature APCs in murine spleen may decrease in uptake of *M.bovis* BCG compared to monocyte derived MΦs and DCs.

### 2.2. Detection of ROS, iNOS and NO of Murine Splenic MΦs and DCs

**Figure 3:**
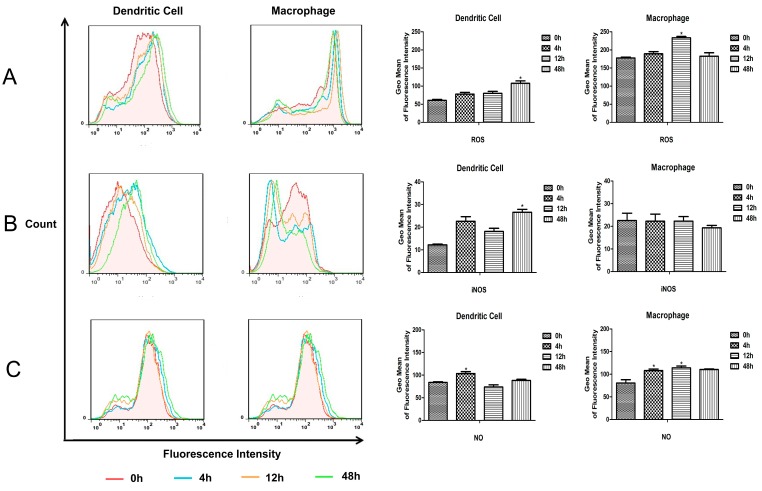
Detection of ROS, iNOS and NO of murine splenic MΦs and DCs. The intracellular ROS (**A**), iNOS (**B**) and NO (**C**) produced in murine splenic MΦs and DCs after *M.bovis* BCG infection were detected. Data depicted represent the mean values ± SEM. Statistical significance was determined by a Student’s *t*-test (* *p* < 0.05).

To study the innate immune responses of murine splenic MΦs and DCs against *M.bovis* BCG, the intracellular reactive oxygen and reactive nitrogen produced in murine splenic MΦs and DCs were detected. The results showed that the ROS and NO level increased in both murine splenic MΦs and DCs, and all the ROS, iNOS and NO level in MΦs was higher than DCs (Figure 3).

### 2.3. Cytokine Secretion from BCG-Infected Murine Splenic MΦs and DCs

**Figure 4:**
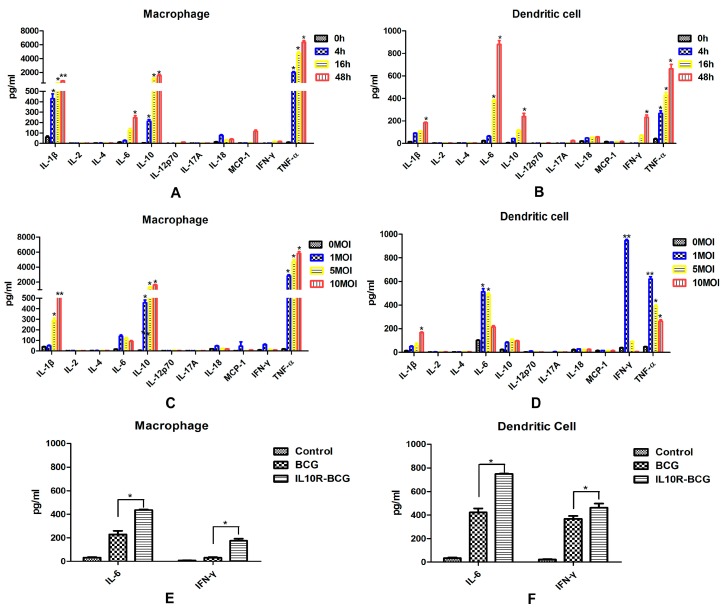
Kinetics of cytokine production following BCG-infected splenic DCs and MΦs. Splenic macrophages (**A**) and dendritic cells (**B**) were infected with BCG at a MOI of 5, and the cell culture supernatants were collected and detected at different time points after infection; Splenic macrophages (**C**) and dendritic cells (**D**) were infected with different MOIs of BCG, and the cell culture supernatants were collected and detected at 16 h after infection; IL-6 and IFN-γ production increased significantly in *M.bovis* BCG-infected MΦs (**E**) and DCs (**F**) when the effect of IL-10 was blocked by soluble IL-10R. Data depicted represent the mean values ± SEM. Statistical significance was determined by a Student’s *t*-test (* *p* < 0.05, ** *p* < 0.01).

In order to analyze the profile and kinetics of cytokine expression of murine splenic MΦs and DCs against *M.bovis* BCG, we collected the cell culture supernatants of murine splenic MΦs and DCs after BCG infection at different time point and detected a serial of inflammatory secreted cytokines. Murine splenic DCs infected with BCG showed enhanced production of IL-6, IL-10, IFN-γ and TNF-α, and IL-6 and TNF-α production was evident already at 16 h, but IL-10 and IFN-γ increased up to 48 h (Figure 4A). A clear different cytokine secretion was observed in MΦs infected with BCG, which also produced high level of IL-6 and TNF-α, whereas low level of IFN-γ was detected, the IL-10 increased obviously, and the MCP-1 was detected at 48 h (Figure 4B). In this study, both murine splenic MΦs and DCs infected with BCG showed increased production of IL-1β, but not significant production of IL-18, and MΦs produced higher levels of IL-1β.

To further study whether different MOIs would affect the cytokine expression profile, different MOIs of BCG were used to infect murine splenic MΦs and DCs cultures. DCs infected with higher doses of BCG produced obviously less cytokines (Figure 4C). However, the cytokine expression profile in macrophages was not obviously changed by a higher infectious dose of BCG, and it increased slightly at a MOI of 5 and 10 (Figure 4D).

The inhibitory role of IL-10 on cytokine production was then investigated. IL-6 and IFN-γ production increased significantly in *M.bovis* BCG-infected MΦs and DCs when the effect of IL-10 was blocked by soluble IL-10R.

### 2.4. Antigen Presenting Assay in Vitro

**Figure 5:**
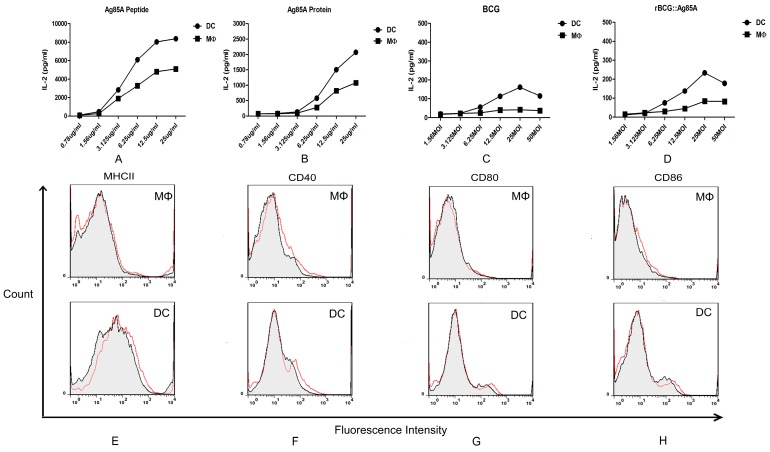
Detection of antigen presenting activity of murine splenic MΦs and DCs *in vitro*. Murine splenic macrophages and dendritic cells were treated with serial diluted Ag85A peptide (**A**), Ag85A protein (**B**), and infected with BCG (**C**), rBCG::Ag85A (**D**), then co-cultured with T hybridoma DE10. The change of cell surface MHCII (**E**), CD40 (**F**), CD80 (**G**) and CD86 (**H**) of murine splenic macrophages and dendritic cells in control group (Black) and BCG infected group (Red).

To investigate and compare the specific antigen presenting activity of murine splenic MΦs and DCs against *M.bovis* BCG antigen, the formation of MΦs and DCs specific Ag85A peptide-MHCII complexes were detected through measuring IL-2 in T hybridoma culture supernatants. The murine splenic MΦs and DCs of C57BL/6 were sorted and treated with serial diluted Ag85A peptide, Ag85A protein, then co-cultured with T hybridoma DE10, specific for a dominant Ag85A peptide p241-160. High levels of Ag85A peptide-MHCII complexes were successfully detected in Ag85A peptide and Ag85A protein treated MΦs and DCs through measuring IL-2 in culture supernatants, and murine DCs had a stronger capacity for presenting Ag85A peptide to specific T hybridoma (Figure 5A,B). When the murine splenic MΦs and DCs were infected with BCG and rBCG::Ag85A, relatively low levels of antigen presenting activity were detected (Figure 5C,D), indicating that the mature APCs in murine spleen decreased in antigen processing of *M.bovis* BCG.

We next detected the expression of cell surface markers involved in antigen presentation and T cell activation. Contrary to murine splenic MΦs, DCs showed high levels of MHC II and co-stimulatory molecules cluster of differentiation 40 (CD40), CD80, CD86, but the molecules did not increase obviously in both mature MΦs and DCs. It suggested that *M.bovis* BCG-infected DCs are likely to function as efficient APCs compared with *M.bovis* BCG-infected MΦs.

## 3. Discussion

The interaction of *M.tb* with MΦs and DCs plays important roles in the pathogenesis of tuberculosis, and it is critical in the process of *M.tb* infection. Both MΦs and DCs have been suggested as being pivotal in triggering the immune effects against mycobacterial infection, and many studies performed in various kinds of animal have suggested that these two cell types are likely to have different roles in immunity against *M.tb* infection [4].

Both MΦs and DCs have been shown to ingest mycobacteria, and activate multiple microbicidal mechanisms. A clear difference of the infectivity of *M.tb* in monocyte-derived MΦs and DCs was observed, which was two-fold higher in DCs compared with MΦs [6]. Following *i.v.* administration in mice, the presence of BCG bacilli in spleen was evaluated by FCM, indicating only 2% of the MΦs population were infected [10]. In this study, we investigated the ability of murine splenic MΦs and DCs to internalize *M.bovis* BCG, and the rBCG-GFP was used to infect sorted murine splenic MΦs and DCs *in vitro*. An obvious difference was observed in the number of inhaled rBCG-GFP, which was greater in murine splenic MΦs compared with DCs, indicating that both murine splenic MΦs and DCs can internalize *M.bovis* BCG, and that murine splenic MΦs have stronger phagocytic activity against BCG than murine DCs. The mature APCs in murine spleen may decrease in uptake of *M.bovis* BCG compared to monocyte derived MΦs and DCs. And the intracellular reactive oxygen and reactive nitrogen produced in murine splenic MΦs and DCs were detected. The results showed that the ROS and NO level increased in both murine splenic MΦs and DCs, and all the ROS, iNOS and NO levels in MΦs were higher than in DCs. These results suggest that the murine splenic MΦs elicit anti-microbial mechanisms for elimination of *M.bovis* BCG more effectively than DCs.

Pro-inflammatory and immunoregulatory cytokines are essential for host immune responses against *M.tb* infection. TNF-α production will lead to granuloma formation and protective immune response in early stages of *M.tb* infection [11,12]. IL-6 is also a pivotal proinflammatory cytokine during acute *M.tb* infection [13]. Immunosuppressive cytokine IL-10 can inhibit the activation of macrophages [14] and the differentiation of dendritic cells [15]. After *M.tb* infection, the proinflammatory cytokines TNF-a, IL-1, and IL-6 were produced mainly by human peripheral blood monocyte-derived macrophages, and Th1-type cytokines IL-12 and IFN-α were secreted almost exclusively from infected monocyte-derived dendritic cells [6]. IL-12 plays a critical role both in the control of bacterial infection and in T cell priming and differentiation. The purified DCs isolated from the spleen 12 h after *in vivo* infection with BCG produced IL-12 p40, but no IL-12 p40 was detected with MΦ and B cells taken from infected mice [10]. In this study, the expression of inflammatory and immunoregulatory cytokines were detected in cell culture supernatants obtained from murine splenic MΦs and DCs infected with *M.bovis* BCG *in vitro*. Murine splenic DCs infected with BCG showed enhanced production of IL-6, IL-10, IFN-γ and TNF-α. A clear different cytokine secretion was observed in MΦs, which also produced high levels of IL-10 and TNF-α, and the IL-6 increased obviously. DCs infected with higher doses of BCG produced obviously lower levels of cytokines. However, the cytokine expression profile in macrophages was not significantly changed by a higher infectious dose of BCG. This suggested that murine splenic MΦs and DCs respond to *M.bovis* in a different fashion. The inflammasome is an intracellular protein complex that activates cysteine protease caspase-1, which induces the maturation and production of a series of pro-inflammatory cytokines, such as IL-1β and IL-18 [16]. Thus, IL-1β and IL-18 are important mediators of the inflammatory response, and provides a good indication of inflammasome activation. In this study, the splenic MΦs produced higher levels of IL-1β, suggesting that MΦs may play a larger role in inducing inflammatory response than DCs.

Although both MΦs and DCs can process and present Ags to T cells, DCs are the primary APCs for naive T cells, and MΦs are not critical for priming T cell responses, but are instead activators of effector and memory T cells [9]. Following *i.v.* administration of live rBCG, DCs but not MΦs acquire APC capabilities for mycobacterial-derived Ags [10], whereas a lot of studies *in vitro* with derived APCs showed that both cell types stimulate activated specific T cells following BCG infection [16]. In this study, we investigated the antigen presenting activity of murine splenic DCs and MΦs against *M.bovis* BCG, using an MHCII-restricted T hybridoma cell line, DE10, specific for an epitope (241–260) from the *M.bovis* BCG Ag85A protein. Ag85A has an important role in cell wall mycolic acid synthesis, and it is associated with the bacterial cell wall and secreted into the surrounding medium [17,18]. High levels of Ag85A peptide-MHCII complexes were successfully detected in Ag85A peptide and Ag85A protein treated MΦs and DCs through measuring IL-2 in culture supernatants. IN addition, murine DCs have a stronger capacity for presenting the Ag85A peptide to specific T hybridomas. These results confirmed that both murine splenic MΦs and DCs are capable of presenting BCG antigen *in vitro*, and murine DCs have a stronger antigen presenting activity than MΦs. We next detected the expression of cell surface markers involved in antigen presentation and T cell activation, and the murine splenic DCs showed a higher level of MHCII and co-stimulatory molecules CD40, CD80, and CD86, it also suggested that *M.bovis* BCG-infected DCs are more likely to function as efficient APCs compared with MΦs.

## 4. Experimental Section

### 4.1. Experimental Animals

Six-week-old female C57BL/6 mice were obtained from VITAL RIVER (Beijing, China). The mice were housed, handled and immunized at the animal biosafety facilities. All work with animals was approved by the institutional animal experimental committee of Yangzhou University.

### 4.2. Bacterial Strains and Culture Conditions

*M.bovis* BCG Pasteur 1173P2 and rBCG-GFP were kindly provided by Dr. Xiaoming Zhang (Institut Pasteur of Shanghai, Chinese Academy of Sciences, Shanghai). rBCG::Ag85A was constructed by our laboratory. *M.bovis* BCG Pasteur 1173P2, rBCG-GFP and rBCG::Ag85A were grown with gentle agitation (80 rpm) in Middlebrook 7H9 medium (BD, Sparks, MD, USA) supplemented with 0.05% Tween 80 and 10% acid-albumin-dextrose-catalase complex (ADC), or on solid Middlebrook 7H10 medium (BD, Sparks, MD, USA) supplemented with 0.05% Tween 80 and 10% oleic acid-albumin-dextrose-catalase complex (OADC).

### 4.3. T Cell Hybridoma and Ags

T cell hybridoma DE10 is specific for immunodominant p241–260 Ag85A peptide and MHC II restricted, and it was kindly provided by Dr. Claude Leclerc (Institut Pasteur, Paris, France) [19]. The Ag85A protein was expressed and purified by our laboratory. The Ag85A peptide was synthesized by SciLight Biotechnology (Beijing, China).

### 4.4. Cell Sorting of Murine Splenic Dendritic Cell and Macrophage

The spleens of C57/BL6 mice were removed and perfused with 400 U/mL collagenase type IV (Invitrogen, Carlsbad, CA, USA) containing 50 μg/mL DNase I (Invitrogen). Single spleen cell suspensions were prepared and APCs were sorted with an autoMACS (Miltenyi Biotec, Bergisch Gladbach, Germany) using CD11c and CD11b cell markers. In this case, spleen cells were first stained with anti-CD11c Microbeads (Miltenyi Biotec) before autoMACS separation leading to a positive cell sample of CD11c^+^ cells (dendritic cell). The negative fraction was further incubated with anti-CD11b Microbeads (Miltenyi Biotec) giving a positive cell fraction containing CD11c^−^CD11b^+^ cells (macrophage). The purity of murine splenic dendritic cells and macrophages were analyzed on FACSCalibur.

### 4.5. Infection Assay Using rBCG-GFP

rBCG-GFP suspensions were added on 1 × 10^6^ murine splenic MΦs and DCs sorted from C57BL/6 mice at a multiplicity of infection (MOI) of 1:1, 1:5, or 1:10, respectively. After 6 h of infection at 37 °C, the cultures were washed with complete RPMI 1640 medium supplemented with 100 mg/mL streptomycin, 100 IU/mL penicillin, and 10% fetal calf serum (FCS) (GIBCO, BRL, Gaithersburg, MD, USA), and cultured for an additional 24 h. The infected cells were analyzed on FACSCalibur, then subsequently analyzed using FlowJo software (BD).

### 4.6. Detection of Intracellular ROS, iNOS and NO

A total of 1 × 10^6^ murine splenic MΦs and DCs were added onto 96-well microplates, respectively. Then the MΦs and DCs cultures were infected with BCG at a MOI value of 1:5, after 6 h of infection at 37 °C, the cultures were gently washed with complete RPMI 1640 medium, and cultured in fresh medium for an additional 24 h. Cell cultures from control group and BCG-infected MΦs and DCs were harvested at different times, and detected by kits according to manufacturers’ instructions.

### 4.7. Detection of Cytokines Produced by Murine Splenic MΦs and DCs

A total of 1 × 10^6^ murine splenic MΦs and DCs sorted from C57BL/6 mice were added onto 96-well microplates, respectively. BCG suspensions were added on MΦs and DCs at MOI values of 1:1, 1:5, or 1:10, after 6 h of infection at 37 °C, the cultures were gently washed with complete RPMI 1640 medium, then cultured for the times indicated in each experiment. Cell culture supernatants from control group and BCG-infected MΦs and DCs were harvested at different times, then detected by mouse inflammation kit and mouse Th1/Th2/Th17 cytokine kit (R&D, Minneapolis, MN, USA), Mouse IL-1β ELISA Set (BD) and Mouse IL-18 ELISA Set (MBL, Woburn, MA, USA) according to manufacturers’ instructions.

### 4.8. Antigen Presentation Assay

For *in vitro* Ag presentation assay, 1 × 10^5^ murine splenic MΦs and DCs sorted from C57BL/6 mice were added onto 96-well microplates, respectively. Then, serially diluted Ag85A peptide, Ag85A protein, BCG and rBCG::Ag85A in complete RPMI 1640 medium were added on 96-well microplates, respectively. After 12 h infection, the MΦs and DCs cultures were fixed with 1% paraformaldehyde for 15 min and washed. A total of 1 × 10^5^ T cell hybridomas DE10 were added to those APC and incubated for 24 h. Supernatants were harvested, frozen, and tested for IL-2 content by sandwich ELISA.

### 4.9. Phenotypic Analysis

A total of 1 × 10^6^ murine splenic MΦs and DCs cultures sorted from C57BL/6 mice were added onto 96-well microplates, respectively. Then, they were infected with BCG at a multiplicity of infection (MOI) of 1:5 as required, after 12 h of infection at 37 °C, the MΦs and DCs cultures were gently washed with medium and collected. We used a combination of FITC-labeled anti-CD11c, PE-labeled anti-CD11b, biotinylated anti-I-Ad, CD86, CD80, CD40, and APC-conjugated streptavidin (BD) was used to reveal biotin conjugates. The labeled cells were analyzed on FACSCalibur for four-color staining analysis and subsequently analyzed using FlowJo software (BD).

### 4.10. Statistical Analysis

All data were expressed as the mean ± SE. Statistical analysis was performed using a Student’s *t*-test. Difference were considered to be significant if *p* < 0.05.

## 5. Conclusions

In conclusion, we compared the function of murine splenic MΦs and DCs in immunity against *M.bovis* BCG. The results indicate that macrophages mainly participate in mycobacteria killing and inducing an inflammatory response, and that dendritic cells are primarily involved in antigen presentation and inducing T cell immune responses.
